# Analysis of therapeutic effects of congenital kyphosis in children due to anterior vertebral bone bridges

**DOI:** 10.3389/fsurg.2024.1369112

**Published:** 2024-08-08

**Authors:** Ke Xu, Cefei Zhang, Bing Xia, Yufeng Zhao, Xiaowei Jiang, Chonghao Li, Weiming Hu, Fuyun Liu

**Affiliations:** Department of Pediatric Orthopedics, The Third Affiliated Hospital of Zhengzhou University, Zhengzhou, China

**Keywords:** congenital kyphosis, vertebral bone bridge, surgical treatment, children, kyphosis

## Abstract

**Objective:**

To investigate the choice of treatment options and long-term orthopedic results of congenital kyphosis in children due to anterior vertebral bone bridges.

**Methods:**

The clinical data of children with congenital kyphosis due to anterior vertebral bridges treated at our center from May 2005 to May 2020 were retrospectively analyzed. We evaluated the clinical features of the deformity, the choice of treatment plan, the change in the Cobb angle of the kyphosis and the improvement of the sagittal trunk deviation before and after treatment and at the final follow-up visit by means of pre-treatment and post-treatment imaging, physical examination and analysis of the case data.

**Results:**

A total of 35 children were included. Clinical follow-up was conducted on a cohort of 5 children, all of whom presented with type Ⅱ congenital kyphosis caused by less than three thoracic anterior bone bridges. The study findings revealed no noteworthy advancement in segmental kyphosis, thoracic kyphosis, lumbar lordosis, and sagittal vertical axis during the final follow-up assessment (*p* > 0.05). In a cohort of 30 pediatric patients who underwent surgical intervention, segmental kyphosis was corrected, with a decrease from an average angle of (40.1 ± 20.5)° to (15.6 ± 9.5)°. Furthermore, significant improvements were noted in segmental kyphosis, thoracic kyphosis, lumbar lordosis, and sagittal vertical axis at the postoperative stage compared to the preoperative stage (*p* < 0.05). Notably, improvements in thoracic kyphosis and lumbar lordosis persisted at the final follow-up visit compared to postoperative (*p* < 0.05).

**Conclusion:**

Type Ⅱ congenital kyphosis in children caused by anterior bony bridges of less than three vertebrae in the thoracic segment can be followed up for a long period, and type Ⅱ/Ⅲ congenital kyphosis caused by anterior bony bridges of the vertebrae in the thoracolumbar, lumbar, and lumbosacral segments requires early surgery.

## 1 Introduction

Congenital kyphosis is a spinal deformity that occurrs in the sagittal plane, resulting from atypical vertebral development during the embryonic phase (1). The classification of vertebral kyphosis is determined by its anatomical structure, which can be categorized into three distinct types: Type I, characterized by insufficient vertebral formation; Type Ⅱ, involving dyssegmentation of the vertebral body; and Type Ⅲ, encompassing a combination of both (2). Congenital kyphosis resulting from vertebral body anterior bony bridges encompasses two distinct types, namely type Ⅱ and type Ⅲ, which exhibit a slightly lower prevalence than congenital scoliosis. Furthermore, this condition displays a higher incidence among females than males, and it predominantly manifests in the thoracolumbar region (3, 4).

Anterior vertebral bone bridges lead to congenital kyphosis in children owing to an imbalance between the anterior and posterior growth of the affected vertebrae. The deformity progresses faster and severe deformity can appear at a young age; therefore, early diagnosis and intervention are required (3). However, there are few reports on the clinical features and early diagnosis of the deformity, and studies on treatment options and long-term correction effects are lacking.

This study retrospectively analyzed the clinical data of 35 children with congenital kyphosis caused by anterior vertebral bridges, compared the deformity Angle of the children before and after treatment, and discussed the early diagnosis and treatment options of congenital kyphosis caused by anterior vertebral bridges.

## 2 Materials and methods

The clinical data of pediatric orthopedic children with congenital kyphosis due to anterior vertebral bone bridge admitted to the Third Affiliated Hospital of Zhengzhou University from May 2005 to May 2020 were retrospectively analyzed, and the diagnosis and treatment were all completed by the same team. The inclusion criteria were as follows: ① anterior vertebral bone bridges leading to type Ⅱ and type Ⅲ congenital kyphosis in children (age <18 years), ② segmental kyphosis ≥20° or progression >5° within six months, ③ no history of previous spinal surgeries, ④ follow-up time ≥3 years, and ⑤ complete imaging and case data. The exclusion criteria were as follows: ① vertebral body malformation (Type I) resulting in congenital kyphosis, ② combined coronal Cobb angle ≥20°, and ③ congenital kyphosis of the cervical spine.

Thirty-five children who met the study criteria were included. Patient demographics, including sex, age, anterior vertebral body bone bridge site, operative time, surgical procedure, osteotomy level, bleeding, type of internal fixation, and complications, were recorded. Changes in segmental kyphosis (SK), thoracic kyphosis (TK) and lumbar lordosis (LL), and improvements in sagittal vertical axis (SVA) were collected before and after surgery and at the final follow-up. The children with clinical follow-up should be followed up every 6 months to 1 year to record the changes of the above angles and the improvement of SVA. The research was carried out in accordance with the guidelines of the Declaration of Helsinki and was approved by the Ethics Committee of the Third Affiliated Hospital of Zhengzhou University.

## 3 Treatment options and surgical techniques

For children with type Ⅱ congenital kyphosis caused by less than three thoracic anterior bone bridges, clinical follow-up was performed after excluding the nervous system and other related malformations. Standing anterior and lateral spinal x-rays were reviewed every 6 months to 1 year, and abnormal segment CT and three-dimensional reconstruction were performed when necessary. The congenital kyphosis caused by bone bridge in thoracolumbar, lumbar and lumbosacral segments was treated by posterior surgery.

The children who underwent surgery were positioned in the prone posture while under general anesthesia. They were subjected to routine sterilization, surgical draping, and continuous neurophysiological monitoring throughout the procedure. A longitudinal incision was made along the affected region of the spinal structure, which exhibited deformities. During the operation, C-arm fluoroscopy was used to localize the deformed vertebral body and pedicle, and the subperiosteal segments above and below the deformed vertebral body that needed to be fused were exposed. Pedicle pins were inserted into the fused segments, and osteotomies were performed according to the surgical plan designed before the operation. ① Schwab Ⅱ osteotomy: removal of the spinous process, part of the vertebral plate and articular process above the intervertebral segment behind the bridge; ② Schwab IV osteotomy: removal of the target pedicle and posterior attachments, neighboring intervertebral discs, and adjacent vertebral cancellous bone; ③ Schwab V osteotomy: complete removal of the target vertebrae and the adjacent upper and lower discs to the adjacent vertebral cancellous bone, under the premise of preserving the anterior vertebral cortex; ④ Schwab Ⅵ osteotomy: the target vertebral body and adjacent upper and lower intervertebral discs are removed with the preservation of the anterior cortex of the vertebral body, and the cancellous bone of the adjacent vertebral body is partially removed during the operation. During osteotomy, it is necessary to ensure safety of the spinal cord and nerve roots. For osteotomies classified as Schwab Ⅳ or higher, if the deformed segment is located in the thoracic vertebrae, it is also necessary to remove the proximal rib head to the rib nodes. Pre-bent titanium rods need to be placed on the contralateral side prior to osteotomy. After the bone block was removed, the titanium rod was connected to the pedicle nail and alternately pressurized to close the osteotomy space and correct the kyphosis. For children with neurological disorders such as spinal cord tethering syndrome and longitudinal spinal fracture, neurological surgery was performed first, followed by kyphosis correction. A brace was worn for 6 months after surgery (5).

## 4 Medical imaging and other auxiliary examinations

Prior to treatment, the following evaluations were conducted: upright/supine (prior to standing) complete spine anterior and side radiographs to measure spinal imaging parameters; computed tomography and three-dimensional reconstruction of deformed segments to clarify spinal anatomy; cranial and spinal MRI to clarify the presence of neurological pathology and intervertebral disc development; lower limb electromyography to evaluate muscle power and sensation; and pulmonary function and echocardiography to assess the child's cardiorespiratory function and rule out additional congenital abnormalities.

Imaging indexes included: ① SK, the angle between the sagittal upper endplate and the lower endplate; ② TK, the angle between the upper endplate of T5 and the lower endplate of T12; ③ LL, the angle between the upper endplate of L1 and the upper endplate of S1; and ④ SVA, the distance between the sagittal C7 vertical line and the vertical line of the posterior-superior corner of the sacrum. A positive value was assigned to the C7 vertical line located in front of the vertical line of the posterior-superior corner of the sacrum, while a negative value was assigned to the opposite scenario. The changes in SK, TK, and LL, and the improvement in SVA were recorded before and after treatment.

## 5 Statistical analysis

The data were analyzed using SPSS26.0 software, and the Wilcoxon signed-rank test was used to compare imaging measurement parameters at the time of diagnosis, 6 months after diagnosis, and at the last follow-up. The paired *t*-test or Wilcoxon signed-rank test was used to compare imaging measurement parameters preoperatively, postoperatively, and at the last follow-up. The mean ± standard deviation (X ± SD) was used to express data that conformed to a normal distribution, whereas the median (P25, P75) was used for data that did not conform to a normal distribution. The test level was set at a=0.05, such that the differences were considered statistically significant at *p *< 0.05.

## 6 Results

A total of 35 children meeting the study criteria were finally included; 21 females and 14 males, all with type Ⅱ/Ⅲ congenital kyphosis, aged 56.9 ± 50.4 months (range, 0.3–192 months). In nine instances (25.7%), the highest point of kyphosis was situated in the thoracic section (T3–T10), affecting 2–6 vertebrae. In nineteen cases (54.3%) were located in the thoracolumbar section (T11–L2), involving 2–5 vertebrae. The lumbar section (L3–L4) was the site in five cases (14.3%), affecting 2–5 vertebrae. Additionally, the lumbosacral section (L5–S2) was involved in two cases (5.7%), affecting 2–4 vertebrae. Type Ⅱ was observed in 20 cases (57.1%), whereas Type Ⅲ was present in 15 cases (42.9%).There were 24 cases (68.6%) of combined spinal cord tethering syndrome, 16 cases (45.7%) of combined longitudinal spinal cord fissure, 8 cases (22.9%) of combined spinal cord cavitation, 2 cases (5.7%) of combined spinal bulge, and 1 case each of combined caudal vertebral deformity, juxtaposition of the ribs, herniation of the cerebellar tonsils, congenital anal atresia, and congenital heart disease.

Of the 35 children in this study, five girls with type II congenital kyphosis caused by less than three thoracic anterior bone bridges were observed at regular follow-up (Table 1). Their ages ranged from 7 to 84 months, with an average of 63.8 ± 33.4 months; The number of vertebrae involved ranged from 2 to 3, with an average of 2.4 ± 0.5 vertebrae; The follow-up period ranged from 56 to 168 months, with an average of 103.0 ± 50.0 months. There were no significant changes (*p* > 0.05) in SK, TK, LL, and SVA between the time of diagnosis and 6 months post diagnosis, as well as between 6 months post-diagnosis and the last follow-up visit (Table 2).

**Table 1:** Clinical data of 5 patients under clinical observation.

Case	Age (months)	Sex	Vertebral bridge site	Type	At diagnosis SK (°)	After diagnosis SK (°)	Follow-up SK (°)	Follow-up (months)
1	84	F	T7–T9	Ⅱ	36	38	27	121
2	7	F	T7–T8	Ⅱ	31	34	43	49
3	60	F	T7–T8	Ⅱ	31	32	34	168
4	84	F	T4–T6	Ⅱ	27	25	27	121
5	84	F	T3–T4	Ⅱ	24	25	26	56

M, male; F, female. After diagnosis, 6 months after diagnosis; Follow-up, last follow-up.

**Table 2:** Comparison of SK, TK, LL, and SVA in 5 patients under clinical observation at diagnosis,6 months after diagnosis and the last follow-up visit.

Parameters	At diagnosis	After diagnosis	Follow-up	*P* (At diagnosis vs. After diagnosis)	*P* (After diagnosis vs. Follow-up)
SK (°)	31.0 (25.5,33.5)	32.0 (25.0,36.0)	27.0 (26.5,38.5)	0.276	0.498
TK (°)	32.0 (23.0,34.0)	33.0 (30.5,40.0)	33.0 (30.0,45.0)	0.480	0.498
LL (°)	27.0 (17.5,37.5)	35.0 (32.0,44.0)	35.0 (30.5,39.0)	0.080	0.343
SVA(mm)	18.0 (2.6,39.5)	17.0 (5.6,19.9)	19.0 (10.0,37.4)	0.343	0.498

After diagnosis, 6 months after diagnosis; Follow-up, last follow-up.

Thirty children underwent transpedicular posterior surgical treatment (Table 3): Schwab Ⅱ osteotomies in 4 cases, Schwab Ⅳ osteotomies in 19 cases, Schwab V osteotomies in 3 cases, and Schwab Ⅵ osteotomies in 4 cases. The average age was 56.9 months (range, 0.3 to 192 months). The average number of vertebrae involved was 3.3, ranging from 2 to 6 vertebrae. The average operative time was 223.3 min on average, with an average intraoperative blood loss of 233.0 ml on average. The fixation of vertebrae averaged 3.0, ranging from 2 to 5 vertebrae. The average postoperative follow-up period was 103.3 months on average, ranging from 37 to 218 months. The correction rate at the last follow-up was 61.1%. There was a significant improvement in postoperative SK, TK, LL, and SVA compared with the preoperative period (*p *< 0.05). TK and LL showed further improvement at the last follow-up compared with the postoperative period (*p *< 0.05) (Table 4).

**Table 3:** Clinical data of 30 patients under operating patients.

Case	Age (months)	Sex	Vertebral bridge site	Type	Osteotomy classification (grades)	Operation time (min)	Volume of bleeding (ml)	Pre.SK (°)	Post.SK (°)	Follow-up SK (°)	Follow-up time (months)	Final correction rate of the SK (%)
1	84	F	T11–L3	Ⅱ	Ⅱ	340	350	41	17	17	96	58.54
2	144	F	L1–L2	Ⅱ	Ⅱ	255	500	22	7	10	49	54.55
3	96	M	L2–L4	Ⅱ	Ⅱ	155	30	32	20	24	191	25.00
4	3	F	T9–L1	Ⅱ	Ⅱ	210	80	33	20	6	150	81.82
5	108	M	L5–S1	Ⅱ	Ⅳ	100	70	24	15	12	108	50.00
6	192	M	L3–L4	Ⅱ	Ⅳ	180	500	33	17	13	76	60.61
7	0.3	F	L2–L4	Ⅱ	Ⅳ	120	20	32	5	3	73	90.63
8	78	F	T11–L1	Ⅲ	Ⅳ	295	500	20	10	15	71	25.00
9	18	F	T12–L3	Ⅱ	Ⅳ	210	100	43	24	15	86	65.12
10	12	M	T4–T7	Ⅲ	Ⅳ	140	200	37	21	25	96	32.43
11	144	F	L2–L3	Ⅱ	Ⅳ	250	600	30	16	19	103	36.67
12	24	M	L1–L2	Ⅲ	Ⅳ	215	200	44	25	22	66	50.00
13	78	F	L1–L5	Ⅱ	Ⅳ	200	300	56	26	23	191	58.93
14	21	M	T12–L1	Ⅲ	Ⅳ	190	200	35	11	16	84	54.29
15	12	F	T11–L1	Ⅲ	Ⅳ	155	50	33	15	31	142	6.06
16	24	F	T6–T8	Ⅲ	Ⅳ	290	250	18	10	22	98	−22.22
17	33	M	T12–L2	Ⅲ	Ⅳ	225	150	34	18	25	38	26.47
18	38	M	C7–T5	Ⅲ	Ⅳ	260	200	21	8	12	93	42.86
19	132	F	T12–L2	Ⅲ	Ⅳ	300	400	54	12	10	107	81.48
20	84	F	T11–L1	Ⅱ	Ⅳ	145	40	28	18	15	149	46.43
21	108	M	T11–L3	Ⅲ	Ⅳ	300	200	14	3	5	89	64.29
22	36	M	T12–L1	Ⅲ	Ⅳ	215	150	57	32	19	59	66.67
23	12	M	T10–L1	Ⅲ	Ⅳ	165	220	15	5	8	122	46.67
24	72	M	L4–S2	Ⅱ	Ⅴ	205	80	83	30	33	218	60.24
25	46	M	L3–L4	Ⅲ	Ⅴ	315	350	27	12	15	60	44.44
26	12	F	T12–L2	Ⅲ	Ⅴ	270	200	65	15	16	37	75.38
27	15	F	T12–L4	Ⅱ	Ⅵ	265	300	72	15	17	83	76.39
28	31	M	L2–L5	Ⅱ	Ⅵ	265	400	88	−12	−18	133	120.45
29	14	F	L1–L4	Ⅲ	Ⅵ	190	150	81	18	21	72	74.07
30	35	F	T12–L1	Ⅱ	Ⅵ	275	200	32	16	18	160	43.75

M, male; F, female. Pre, preoperative; Post, postoperative; Follow-up, last follow-up.

**Table 4:** Comparison of SK, TK, LL, and SVA in 30 operated children with preoperatively, postoperatively, and at the last follow-up visit.

Parameters	Pre	Post	Follow-up	Pre vs. Post		Post vs. Follow-up	
				*t*	*P*	*t*	*P*
SK (°)	40.1 ± 20.5	14.9 ± 8.7	15.6 ± 9.5	6.650	<0.001	−0.582	0.565
TK (°)	14.5 ± 12.3	18.9 ± 5.4	23.0 ± 5.5	−2.606	0.014	−3.584	0.001
LL (°)	1.0 ± 34.0	10.1 ± 18.0	19.4 ± 13.6	−2.689	0.012	−2.870	0.008
SVA(mm)	20.3 ± 26.6	9.6 ± 4.9	8.8 ± 5.5	2.344	0.026	0.799	0.431

Pre, preoperative; Post, postoperative; Follow-up, last follow-up.

Seven of the 30 children treated surgically were treated with vertebral resection combined with pedicle nails or hooks while preserving the anterior vertebral cortex. The average age of these children was 32.1 ± 21.7 months (ranging from 12 to 72 months), and they had involvement of 3.4 ± 1.1 vertebrae (ranging from 2 to 5 vertebrae). The duration of the operation was 255.0 ± 43.1 min, with an intraoperative blood loss of 240.0 ± 114.2 milliliters. The fixation was performed on an average of 2.9 ± 0.9 vertebrae (ranging from 2 to 4 vertebrae). The mean postoperative follow-up period was 109.0 ± 64.1 months (ranging from 37 to 218 months), and the correction rate at the last follow-up was 76.4%. Postoperative SK and TK showed significant improvement compared with the preoperative period (*p *< 0.05); TK and LL showed further improvement at the last follow-up compared with the postoperative phase (*p *< 0.05) (Table 5).

**Table 5:** Comparison of SK, TK, LL, and SVA in 7 patients who treated with vertebral resection combined with pedicle nails or hooks while preserving the anterior vertebral cortex with preoperatively, postoperatively, and at the last follow-up.

Parameters	Pre	Post	Follow-up	*P* (Pre vs. Post)	*P* (Post vs Follow-up)
SK (°)	72.0 (32.0,83.0)	15.0 (12.0,18.0)	17.0 (15.0,21.0)	0.018	0.233
TK (°)	14.0 (8.0,18.0)	19.0 (17.0,28.0)	29.0 (25.0,34.0)	0.027	0.034
LL (°)	−28.0 (−38.0,40.0)	−3.0 (−12.0,20.0)	29.0 (19.0,33.0)	0.070	0.028
SVA (mm)	−15.0 (−18.0,24.0)	7.0 (5.0,13.0)	12.0 (4.0,13.0)	0.128	0.672

Pre, preoperative; Post, postoperative; Follow-up, last follow-up.

**Figure 1:**
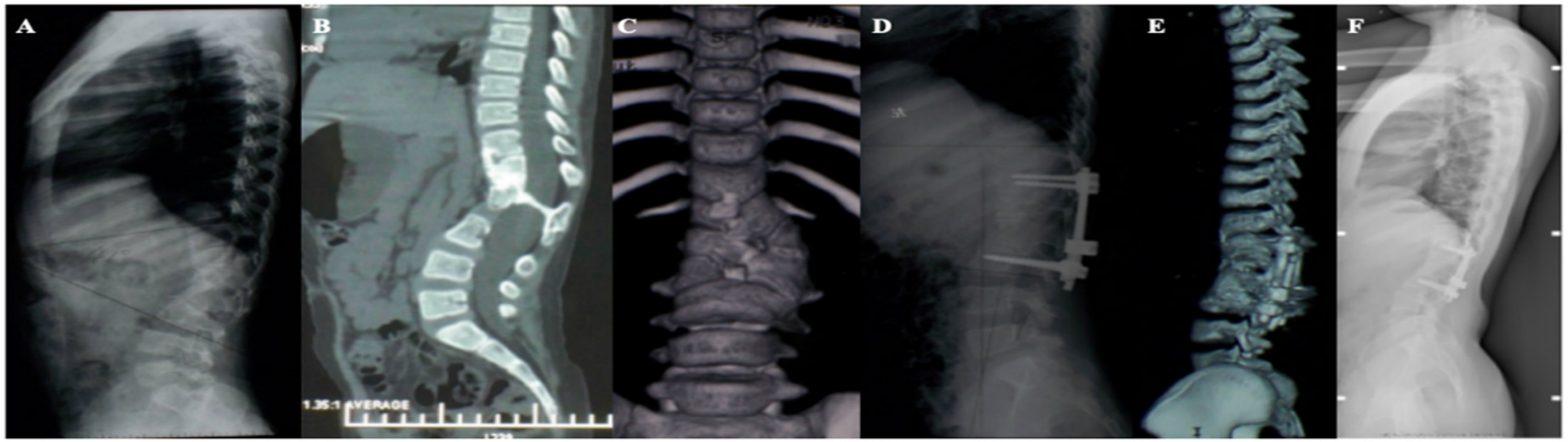
(**A–C**) A 7-year-old girl, congenital kyphosis due to anterior bone bridge of T11–L3 vertebrae, type III, type I longitudinal spinal cord fracture, segmental kyphosis Cobb angle 41°; (**D,E**) schwab grade II osteotomy combined with pedicle screw internal fixation at T12-L3 after resection of intraspinal bony spines, postoperative segmental kyphosis Cobb angle 17°, correction rate 58.5%; (**F**) review at 8 years postoperatively, segmental kyphosis Cobb angle 17°, correction rate without loss.

**Figure 2:**
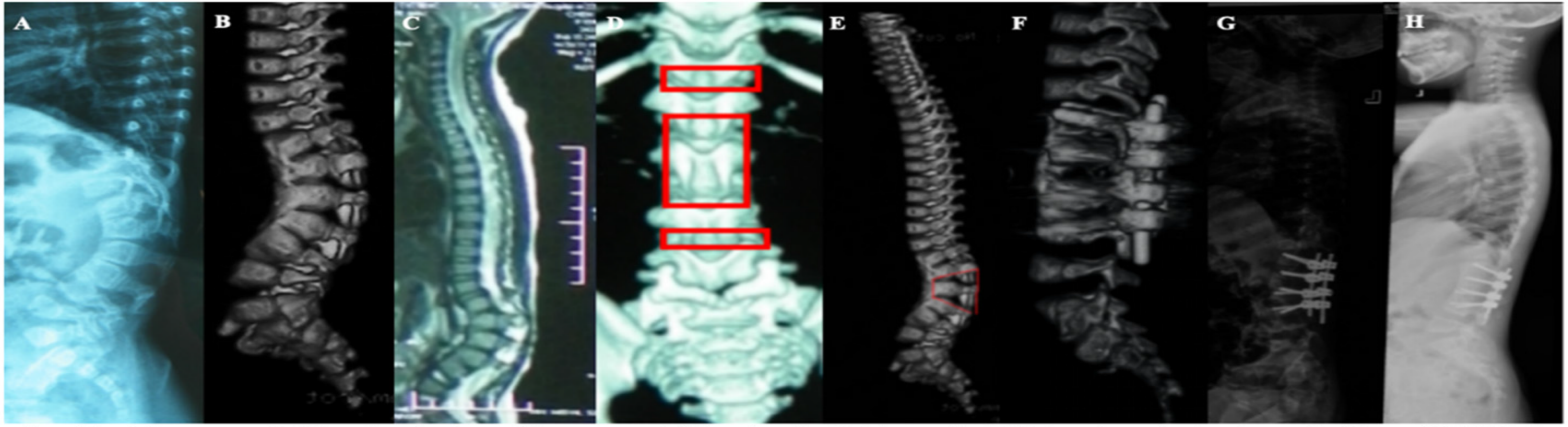
(**A–C**) A 15-month-old girl, congenital kyphosis, type II, segmental kyphosis Cobb angle 72° due to anterior bone bridge of T12–L4 vertebral body; (**D,E**) preoperative design of surgical plan, schwab VI osteotomy of L2 with preservation of anterior bone cortex of the vertebral body, and schwab II osteotomy of T12–L1, L3–L4, and simultaneous combination of internal fixation with pedicle screws; (**F,G**) postoperative review, the anterior cortex of the vertebral body was preserved without fracture, and the kyphosis correction was satisfactory, with a segmental kyphosis cobb angle of 15°, a correction rate of 79.2%, but there was a proximal junctional kyphosis cobb angle of 30°; (**H**) Postoperative review 7 years after the operation the segmental kyphosis Cobb angle of 17° was found to be corrected at a rate of 76.4%, and the proximal junctional kyphosis had disappeared.

**Figure 3:**
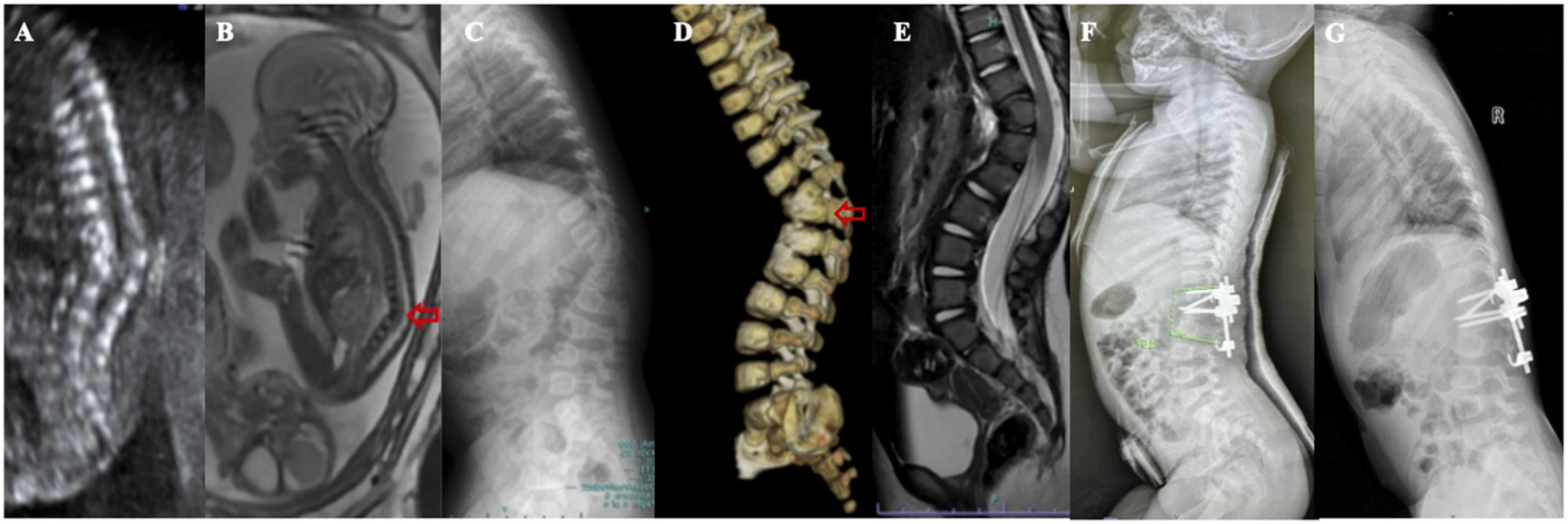
(**A**) A 22-week female fetus, fetal ultrasound showed obvious kyphosis deformity of the thoracolumbar segment, with localized vertebral echogenicity, disordered alignment, varying sizes, and varying widths of the vertebral interspaces; (**B**) fetal MRL showed congenital kyphosis in the thoracolumbar segment, with vertebral body morphology irregularities; (**C–E**) the girl was perfected at the age of 1 year, and the anterior bony bridge in the vertebral body of T12–L2 resulted in congenital kyphosis with a half-vertebral body to the left of the interval between T12–L1 and a 6-fractional lumbar vertebrae, SK72°; (**F**) schwab grade IV osteotomy was performed in L1 and grade II osteotomy in L2. Plaster external fixation was performed one after surgery, SK15°, kyphotic correction rate of 79.2%; (**G**) Reexamination 37 months after surgery showed SK16° and almost no loss of correction rate.

One child developed unilateral numbness of the lower limbs after surgery, and was treated with methylprednisolone and nutritive nerve drugs, and the symptoms were relieved after 5 days. None of the operated children had complications such as internal fixation infections, loosening and breakage, and there was no pseudoarthrosis formation. Three typical cases (Figures 1–3) are attached.

## 7 Discussion

With anterior growth restriction of the deformed vertebrae due to the formation of bony bridges and fascia, and continued growth of the posterior vertebrae and appendages, kyphosis will continue to progress with age. The rate of progression of congenital kyphosis due to anterior vertebral bridges is closely related to the site, extent, and number of bridges involved, whether or not they are accompanied by poor vertebral body formation, and the growth potential of the posterior vertebrae (6). To maintain trunk equilibrium, the upper and lower vertebrae of the curved part are often accompanied by functional compensatory reverse curvature. If the segmental curvature is not corrected in a timely manner, this functional compensatory curvature gradually progresses to a structural compensatory curvature with the growth of the spine. Anterior bony bridges of the thoracic segmental vertebrae lead to type II congenital kyphosis in children with short spinal segments (2–3 vertebrae), no more than 3 vertebrae, late onset of symptoms, mild and slow progression, with an average rate of progression of 2°–6.5°/year. The sagittal position is very mildly affecting TK, LL, and SVA, and is not susceptible to structural compensatory curvature (7). Anterior bone bridges in the thoracolumbar, lumbar, and lumbosacral vertebrae involve long vertebral segments (2–5 vertebrae) and gravity factors, causing spinal deformities to progress more rapidly in children than in the thoracic segment (8). Studies have reported that congenital kyphosis due to anterior bony bridges of the vertebrae in the thoracolumbar segment can progress by 5° to 10° per year during the growth spurt, predisposing to sagittal imbalance of the spine with structural compensatory curvature of adjacent segments (9). Therefore, the anterior bone bridge of thoracolumbar, lumbar and lumbosacral vertebrae in children with congenital kyphosis requires early diagnosis and early treatment.

A standardized timing for surgery in children with congenital kyphosis has not been established but typically depends on the extent of the deformity, the rate of progression, and the overall physical condition of the child, which collectively determines the best time of action. It has been suggested that children with congenital spinal deformities should be operated on as early as possible (before the age of 10 years) when they are older than 1 year or weigh more than 10 kg (10, 11). The study suggests that neonatal kyphosis can be corrected at the same time as the spinal cord is repaired in children with severe spinal cord enlargement (12). During the study, two infants (under 12 months of age) with severe spinal neurological deformities underwent simultaneous surgery to correct spinal kyphosis, yielding satisfactory outcomes. Additionally, four children underwent surgery at a later stage (>10 years old) due to delayed consultation, and the orthopedic results remained acceptable during the final follow-up appointment. With early surgery, children had a low degree of spinal deformity, good flexibility of compensatory curvature, low surgical difficulty, low bleeding, low risk of nerve injury, short fusion segments, and good orthopedic results.

In children with congenital kyphosis due to anterior vertebral bone bridges, osteotomy and orthopedic treatment combined with internal fixation changed the area of spinal deformity from an unstable trapezoidal structure to a stable rectangular structure; at the same time, fusion of the intervertebral implants on the side of the kyphosis was performed, and the orthopedic effect was satisfactory (13). In 2014 Schwab et al. categorized into spinal osteotomies and orthopedics into six levels according to the anatomy of the spine (14). Schwab Ⅱ osteotomy is suitable for patients with arcuate kyphosis who have short spinal segments and underdeveloped intervertebral discs because of the involvement of the anterior bony bridges of the vertebral bodies. Compared with three-column osteotomy, this level of osteotomy is simpler and safer, and it is acceptable for the correction of mild kyphosis in children; however, it is ineffective in the long term for the treatment of severe kyphosis in children (15). The study included the administration of a simple Schwab II osteotomy combined with pedicle screws to a cohort of four children, following an average postoperative monitoring period of 121.5 months, and the SK deformity was rectified from an initial angle of 32.5° to 13.5°, yielding a correction rate of 58.5%. Shi et al. treated 38 cases of congenital kyphosis with Schwab grade Ⅳ osteotomy combined with internal fixation, and the average duration of postoperative follow-up was 38.8 months, and the SK was successfully reduced from 49.5° to 6.8°, achieving a correction rate of 86.3% (15). During this study, a total of 19 children were treated with Schwab Ⅳ level osteotomy combined with pedicle nail. The average duration of follow-up after the operation was 97.4 months, and the SK was successfully reduced from 33.1° to 16.3°, achieving a correction rate of 50.8%.

Schwab V and Ⅵ osteotomies can be performed in all directions while controlling both the anterior and posterior portions of the spine, providing better clinical orthopedic results than other osteotomies (8). Wang et al. performed Schwab Grade V osteotomy combined with internal fixation in 24 children with congenital kyphosis, the average follow-up period was 56.9 months, and they achieved a correction of the SK from 87.3° to 17.6°, with an average correction rate of 79.8% (16). Shi et al. performed Schwab Ⅵ osteotomy combined with internal fixation in 17 children with severe rigid congenital kyphosis, the average follow-up period was 30.8 months, and the SK was reduced from 102.9° to 43.5°, achieving a mean correction rate of 57.7% (17). For severe congenital kyphosis caused by anterior vertebral bone bridges in young children, Schwab V or Ⅵ level osteotomy combined with internal fixation of the pedicle nail or hook was performed under the premise of preserving the anterior vertebral bone cortex. The children were small in age, with great bone toughness, and when the convex side of the osteotomy was pressurized to close the osteotomy gap, the anterior vertebral bone cortex formed “a hinge” structure, which was not easy to fracture, making it safer and more effective in children. In this research, we employed this surgical procedure combined with internal fixation to manage 7 young patients with an average age of 32.1 months and an average monitoring duration of 109.0 months, and the correction of SK improved from 72.0° before the operation to 15.0° immediately after the surgery, and 17.0° during the final follow-up, and the correction rate at the final follow-up was 76.4%, and no complications were observed during the perioperative or follow-up periods. High-grade osteotomy also has some disadvantages. The wider scope of osteotomy is more demanding for the operator, the intraoperative blood loss increases, and the probability of paraplegia and other neurological complications increases significantly; therefore, it is necessary to fully consider the advantages and disadvantages when choosing a surgical procedure (17–19). Furthermore, no instances of proximal junctional kyphosis were observed in the operated children during the final follow-up, which could potentially be attributed to the moderate correction of kyphosis through osteotomy, which was performed to prevent excessive correction that could lead to sagittal imbalance of the spine.

## 8 Limitations

The limited sample size of children in this study posed constraints on the ability to compare and statistically analyze clinical follow-up outcomes across various thoracic segments. Furthermore, a subset of cases had a relatively brief follow-up duration, potentially leading to alterations in the corrective measures of the spinal curvature during subsequent follow-up periods. Expanding the sample size and extending the duration of follow-up would facilitate a more comprehensive assessment of the long-term surgical outcomes.

## 9 Conclusion

Children with type Ⅱ congenital kyphosis caused by less than three thoracic anterior bone bridges can be followed up for a long time, while those with type Ⅱ/Ⅲ congenital kyphosis caused by thoracolumbar, lumbar, and lumbosacral anterior bone Bridges require early surgery. For children with severe congenital kyphosis caused by anterior vertebral bridge, Schwab grade V and Ⅵ osteotomy combined with pedicle nail/hook therapy can be performed by posterior approach to preserve the anterior vertebral cortex. The fixation segment is short and the postoperative effect is accurate.

## Data Availability

The original contributions presented in the study are included in the article/Supplementary Material, further inquiries can be directed to the corresponding authors.
